# Interleaved Sparse–Dense Scanning for Low-Latency Obstacle Detection and 3D Mapping on an Embedded Robotic Platform

**DOI:** 10.3390/s26092732

**Published:** 2026-04-28

**Authors:** Syed Khubaib Ali, Ali A. Al-Temeemy, Pan Cao

**Affiliations:** 1 School of Physics, Engineering and Computer Science, University of Hertfordshire, Hatfield AL10 9AB, UK; a.al-temeemy@herts.ac.uk (A.A.A.-T.); p.cao@herts.ac.uk (P.C.); 2 Department of Laser and Optoelectronics Engineering, College of Engineering, Al-Nahrain University, Baghdad 10072, Iraq; 3 Department of Electrical Engineering and Electronics, Faculty of Science and Engineering, University of Liverpool, Liverpool L69 7ZX, UK

**Keywords:** LiDAR, embedded robotics, obstacle avoidance, 3D mapping, sparse scanning, dense scanning, reactive navigation, point cloud, time-of-flight sensing

## Abstract

LiDAR is widely used in robotics because it provides reliable range data for navigation and mapping. On a small embedded robot, however, there is a practical conflict between scan resolution and reaction speed. Dense scans provide better environmental detail, but they take too long for fast obstacle avoidance, whereas sparse scans are faster but can miss obstacles if the spacing between adjacent rays is too large. This paper presents an Interleaved Sparse–Dense Scanning method for a servo-actuated single-point time-of-flight LiDAR mounted on an embedded mobile robot. A dense nested pan–tilt sweep is used for three-dimensional mapping, while a sparse forward scan is inserted between dense rows for obstacle detection and motion control. A geometric model is derived to relate sensing range, beam spacing, and minimum detectable object width. That model is then linked to zone-based safety constraints and to the distance the robot can travel before the next obstacle update. For the robot used in this study, the resulting sparse configuration is a 7-point forward scan over a 180° field of view. Experiments in a real indoor environment showed that this configuration reliably detected target blocking obstacles and reduced decision latency by 6.2 times compared with waiting for a complete dense scan before each navigation update. The proposed method provides a practical balance between reactive obstacle avoidance and useful 3D mapping on a low-cost embedded platform, while making the system’s timing and safety limits explicit.

## 1. Introduction

### 1.1. Background and Motivation

Light Detection and Ranging (LiDAR) has become one of the main sensing technologies used in robotics, mapping, surveying, and autonomous navigation. By measuring the time it takes for a laser pulse to travel to a target and return, LiDAR systems provide range data that can be converted into spatial coordinates and used to describe the surrounding environment [1,2]. Because these systems operate at optical wavelengths, they can provide fine spatial detail, which is one reason why they are attractive for robotic perception [3,4].

In mobile robotics, LiDAR is commonly used for obstacle detection, localisation, and Simultaneous Localisation and Mapping (SLAM). Dense scans can support detailed environmental reconstruction and accurate map building, and are widely used in systems such as scan matching, occupancy-grid mapping, LOAM (LiDAR Odometry and Mapping), and related SLAM pipelines [5,6,7,8,9,10,11]. The difficulty in a small embedded robot is that dense scanning takes time. On a platform with a mechanically actuated sensor, each additional scan point adds a delay before the next navigation decision can be made. That delay may be acceptable for mapping, but it becomes a real problem for reactive obstacle avoidance.

Sparse scanning reduces this delay, but it introduces another problem. If the gap between adjacent scan rays is too large, a narrow or small obstacle can lie between them and go undetected. The design problem is therefore not simply a choice between a fast scan and a dense scan. The scan must be fast enough to support navigation but also dense enough to remain safe.

This issue becomes particularly clear on a servo-actuated embedded platform. As the scan step size is reduced, spatial density improves, but scan time increases sharply. That trade-off affects not only map quality, but also obstacle-response latency and safe robot motion. The present work addresses this problem by presenting an Interleaved Sparse–Dense Scanning method. The system does not rely on one scan mode for everything. Instead, it uses a sparse forward scan for obstacle detection and a denser nested pan–tilt sweep for 3D mapping. These two modes are interleaved, so the robot can react quickly while still collecting enough data to build a useful environmental model.

### 1.2. Related Work

The recent research works related to the scope of the presented work focuses on developing hardware architecture for sensing systems, reactive navigation methods, SLAM-based mapping systems, scan scheduling, and adaptive sensing.

The literature closest to the present work uses a single-range sensor, together with mechanical scanning, to recover additional spatial dimensions. Al-Temeemy and Spencer showed that a single laser distance sensor provides only one dimension of the scanned object, while the remaining two dimensions can be acquired by positioning the sensor in pan and tilt using a controlled scanning unit [12]. This is important for the present work because it directly supports the hardware idea used here: low-cost 3D scanning is possible with a single-point sensor and a pan–tilt mechanism. The same work also explains why such a design is useful. Traditional LiDAR systems are expensive, and their prototypes were developed specifically to provide high-scanning capability at lower cost [12].

Reactive navigation methods such as the Vector Field Histogram (VFH) [13] and the Dynamic Window Approach (DWA) [14] are designed to generate motion decisions quickly from range data. These methods are effective for local obstacle avoidance but do not build a persistent 3D model of the environment. In that sense, they solve a different problem.

Many mobile robots use a level-mounted continuous-rotation LiDAR to produce a horizontal scan slice for 2D obstacle sensing and occupancy-grid mapping. This is highly effective for planar navigation and 2D SLAM, as seen in systems based on occupancy-grid mapping and scan matching [5,8,9]. More advanced mapping systems, including LOAM (LiDAR Odometry and Mapping) and later LiDAR odometry variants, produce richer spatial reconstructions and stronger localisation performance [10,15,16,17,18]. Related work on 3D point-cloud modelling and registration also shows the broader utility of dense point sets for robotics and scene understanding [19,20,21]. More recent tightly coupled LiDAR–inertial and optimisation-based systems further demonstrate the performance gains available when richer sensing and greater computational resources are available [22,23,24]. Full autonomous-driving stacks also illustrate the extent to which dense sensing and high compute budgets can improve perception and navigation performance in larger-scale deployments [25,26,27,28,29,30].

A further body of work on LiDAR systems for autonomous vehicles and 3D mapping provides additional context [31,32,33,34,35,36]. Bajcsy’s classic formulation of active perception emphasised that sensing itself can be directed by task needs rather than treated as a fixed, passive input [37]. More recent work has explored adaptive scan-distance scheduling inside SLAM pipelines to reduce computation while maintaining useful map quality [38], as well as adaptive foveated scanning for depth sensors to allocate measurements non-uniformly according to scene requirements [39]. Work on actuated 3D sensors also shows the flexibility of mechanically moved depth sensors in robotic systems [40]. These studies are relevant because they show that sensing density, spatial coverage, and computational cost can be traded against one another. However, they do not directly address the embedded obstacle-detection problem considered here, in which sparse forward sensing, dense 3D mapping, and safe forward motion must be explicitly linked on a mechanically actuated single-beam platform.

### 1.3. Our Contributions

The main technical contribution is not merely the use of both sparse and dense scans. The main contribution is a configuration-specific design framework that links three quantities in one analysis: obstacle detectability, scan-update latency, and safe forward motion. In other words, the paper turns the choice of sparse scan density from an empirical tuning problem into an explicit engineering design problem for the target platform.

This distinction matters because the present hardware is not a high-rate multi-beam LiDAR system. Unlike previous work on scan scheduling or adaptive LiDAR sampling, which generally assumes high-rate multi-beam LiDAR sensors and focuses on computational optimisation within SLAM pipelines, this study addresses a mechanically actuated single-beam LiDAR platform where actuator motion dominates sensing latency. In such systems, each additional sensing direction introduces a mechanical time cost that directly affects navigation reaction time. The proposed method, therefore, focuses on an Interleaved Sparse–Dense Scanning (ISDS) strategy, in which a sparse forward scan is used for rapid obstacle detection while dense scans are used for mapping. In addition, a geometric detection model is introduced to link beam count, sensing range, and minimum detectable obstacle width with robot motion constraints. This provides a configuration-specific framework that explicitly connects sensing density, detection reliability, and safe robot motion on a low-cost embedded robotic platform [38,39,40]. The resulting problem is therefore different from generic adaptive LiDAR sampling. On this platform, each additional sensing direction incurs a direct mechanical time cost, which affects not only map density but also braking margin and the safe travel distance before the next obstacle update.

This fills a specific gap: it provides an embedded-platform method in which sparse forward scanning and dense mapping are interleaved, while the sparse scan itself is selected using a geometric detection model tied directly to motion safety and timing.

The main contributions of this work are as follows:

- A practical sensing architecture that interleaves sparse forward scans with dense mapping sweeps on a single embedded LiDAR platform.
- A formal geometric-detection model linking beam count, sensing range, and minimum detectable obstacle width for the target blocking obstacle class.
- A zone-based safety analysis showing how detectability changes with distance and how it relates to braking margin.
- A motion-coupled timing model linking scan cycle time, robot speed, and safe forward travel distance.
- Experimental validation showing that a 7-point sparse scan gives the best balance between safety and response time on the current platform.

The rest of the paper is organised as follows. Section 2 presents the robotic system and modelling framework. Section 3 presents the Interleaved Sparse–Dense Scanning method and its analysis. Section 4 presents the experimental results and discussion. Section 5 concludes the paper.

## 2. Systems and Modelling

### 2.1. System Definition and Parameters

**Figure 1:**
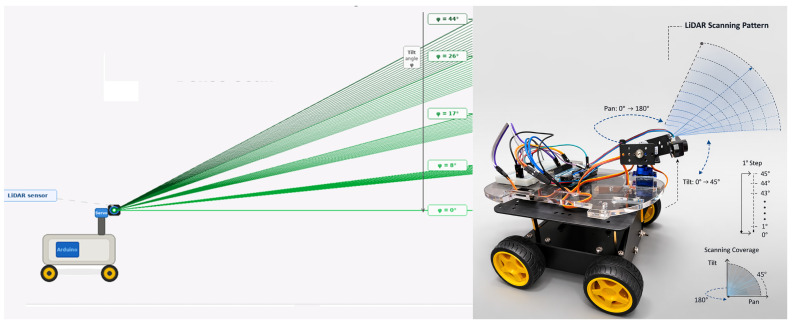
Robotic platform equipped with a servo-actuated LiDAR scanning unit. The sensor is mounted on a pan–tilt mechanism that performs horizontal sweeps from 0° to 180° while incrementally adjusting the vertical tilt angle from 0° to 40°. Arrows indicate the direction of LiDAR beam sweeps; the shaded region shows the resulting scanning coverage area. This configuration enables dense 3D environmental scanning while supporting the interleaved sparse scan used for obstacle detection.

**Figure 2:**
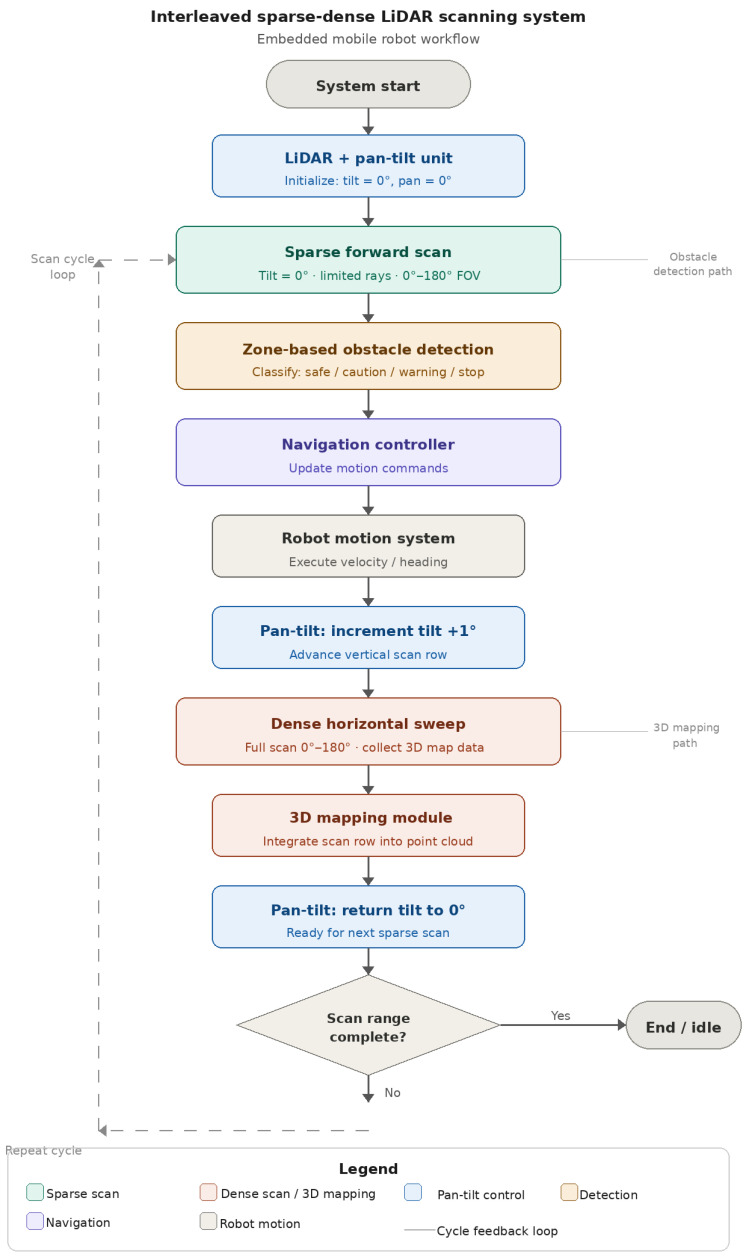
Workflow of the proposed Interleaved Sparse–Dense Scanning (ISDS) system. A sparse forward scan at zero tilt is used for obstacle detection and navigation decisions, while dense horizontal sweeps at successive tilt angles (0°–180°) are used for 3D mapping. Arrows indicate process flow direction; the legend within the figure defines all block types. After each sparse scan, the robot motion is updated, and the tilt angle is incremented before the next dense scan row is acquired. The cycle repeats until the maximum tilt angle is reached.

We consider a robotic sensing platform, consisting of a differential-drive robot, a LiDAR sensor, a servo-based scanning mechanism, an onboard controller, and a host computer used for visualisation and analysis. The sensing task is divided into two parts. A sparse forward scan is used for obstacle detection and immediate motion decisions. A dense nested pan–tilt sweep is used to collect the point cloud needed for the three-dimensional mapping. The overall workflow is shown in Figure 1 and Figure 2. This structure is useful on a small robot because it allows mapping and obstacle detection to support each other without forcing the robot to wait for a complete 3D scan before moving again.

### 2.2. Modelling

Each LiDAR measurement *r* acquired at pan angle θ and tilt angle ϕ is transformed into Cartesian coordinates for point-cloud generation. In the coordinate convention used here, the *y*-axis points forward from the robot, the *x*-axis spans the lateral direction, and the tilt angle ϕ is measured downward from the positive vertical axis of the scan frame [3,21]. Under this convention,

(1) x=rsin(θ)sin(ϕ),

(2) y=rsin(θ)cos(ϕ),

(3) z=rcos(ϕ).

The resulting (x,y,z) points are transmitted to the host computer and visualised in MATLAB (R2023b, MathWorks, Natick, MA, USA) and GNU Octave (version 11.1.0, Free Software Foundation).

The horizontal and vertical scan steps control the density of the reconstructed point cloud. If the horizontal step size is denoted by $\Delta \theta_{h}$ (horizontal angular step used in dense scanning) and the vertical step size by Δϕ (vertical angular step used in dense scanning), then the approximate point spacing at sensing range *R* is

(4) $$\Delta s_{h}\approx R\Delta \theta_{h},$$

(5) $$\Delta s_{v}\approx R\Delta \phi ,$$

where θ (pan angle) and ϕ (tilt angle) are both expressed in radians. The horizontal step controls the spacing between adjacent points in each scan row. The vertical step controls the spacing between adjacent rows. Smaller steps improve resolution, but they increase the total scan time. For the present hardware, a horizontal step of 5° was selected as the practical dense-scan setting because it provided a good compromise between spatial detail and sweep time. The vertical tilt range used for mapping was limited to 0° ≤ϕ≤ 40°.

The key question for the sparse scan is how many rays are needed to reliably detect a blocking obstacle. Consider a forward field of view Θ (total field of view of the sparse scan, in radians) covered by *N* (number of sparse forward scan rays) uniformly spaced rays. The angular spacing between adjacent rays is

(6) $$\Delta \theta =\frac{\Theta }{N-1},$$

where Δθ denotes the angular spacing between adjacent sparse rays. At the effective sensing range $R_{\mathrm{eff}}$ (usable sensing distance after subtracting measurement error), this spacing corresponds to a physical gap in the environment. Under the simplified geometric model used here, the minimum detectable obstacle width is

(7) $$W_{\min}=R_{\mathrm{eff}}\Delta \theta =\frac{R_{\mathrm{eff}}\Theta }{N-1}.$$

For a 180° forward scan, Θ=π, so

(8) $$W_{\min}=\frac{\pi R_{\mathrm{eff}}}{N-1}.$$

Equivalently, for a target obstacle width *W*, the minimum beam count must satisfy

(9) $$N\geq \frac{\pi R_{\mathrm{eff}}}{W}+1.$$

#### Minimum Detection Guarantee

Assume a static scene, a 180° forward scan, and *N* uniformly spaced rays. Let $R_{\mathrm{eff}}$ denote the effective sensing range and let *W* denote the projected width of a convex obstacle. Then, any such obstacle is guaranteed to intersect at least one scan ray if

(10) $$N\geq \frac{\pi R_{\mathrm{eff}}}{W}+1.$$

Adjacent scan rays subtend an angular gap Δθ=π/(N−1). At range $R_{\mathrm{eff}}$, the corresponding physical separation is $R_{\mathrm{eff}}\Delta \theta =\pi R_{\mathrm{eff}}/\left(N-1\right)$. A convex obstacle narrower than this separation can lie entirely between adjacent rays and may be missed. Therefore, guaranteed detection requires $W\geq \pi R_{\mathrm{eff}}/\left(N-1\right)$, which rearranges to Equation (9).

Equivalently, the minimum guaranteed detectable obstacle width for a given beam count is $W_{\min}=\pi R_{\mathrm{eff}}/\left(N-1\right)$.

These assumptions hold when obstacles are stationary and present a convex cross-section to the scanning plane. In practice, concave shapes, partially occluded objects, or moving targets may violate these conditions. For example, a U-shaped obstacle or a narrow pole partially hidden behind another object may not subtend a sufficient projected width to guarantee ray intersection. These cases represent known limitations of the present geometric model and are discussed further in Section 4.4.

## 3. Interleaved Sparse–Dense Scanning Method and Analysis

### 3.1. Method

The proposed method separates two sensing objectives that are often forced into one scan on small robots. A dense nested pan–tilt sweep is used for three-dimensional mapping, while a sparse forward scan is used for obstacle detection and motion control. The key idea is not simply to alternate scans arbitrarily. Rather, the sparse scan is deliberately inserted between dense rows so that obstacle information is refreshed before the robot continues advancing.

The dense scan used for mapping is executed as a nested pan–tilt sweep. At each vertical tilt angle ϕ, the sensor performs a full horizontal sweep from 0° to 180°. Once that horizontal row is complete, the tilt angle is incremented, and the next horizontal sweep begins. In the present system, the tilt angle is limited to the interval 0° ≤ϕ≤ 40°.

The sparse scan is inserted between dense rows. After one complete horizontal sweep is finished, a rapid forward scan is executed at ϕ = 0°. This sparse scan is not meant for map detail. Its purpose is to check whether the forward path is blocked and to update the robot’s motion state before the next dense row begins. This ordering is important. The sparse scan does not wait until the entire 3D scan is complete. It is inserted after each horizontal sweep so the robot can react much sooner.

In effect, the system trades some temporal continuity in dense mapping for a substantial gain in obstacle-response frequency. The overall operation workflow is shown in Figure 2.

### 3.2. Analysis

The effective sensing range $R_{\mathrm{eff}}$ means the usable sensing distance after sensor error has been subtracted from the nominal design range. In this study, the nominal design range was 60 cm, and the mean absolute measurement error was 2.83 cm, so

(11) $$R_{\mathrm{eff}}=60-2.83=57.17\;\mathrm{cm}.$$

In this study, the mean absolute error of 2.83 cm is treated as a constant offset for simplicity. In practice, time-of-flight measurement error varies with target distance, surface reflectivity, and ambient lighting conditions [1,41]. A more detailed error model, accounting for distance-dependent noise characteristics, would refine the estimate of $R_{\mathrm{eff}}$ across operating ranges and is identified as a direction for future work.

The target blocking obstacle width is the minimum obstacle width considered large enough to obstruct the robot’s path and therefore require reliable detection. For the present platform, this width was set to 30 cm, representing a blocking obstacle of comparable scale to the robot body width and the safety margin used in the navigation logic. Substituting $R_{\mathrm{eff}}=57.17$ cm and W=30 cm into Equation (9) gives

(12) $$N\geq \frac{\pi \times 57.17}{30}+1\approx 6.99.$$

This leads to a minimum practical beam count of 7.

**Figure 3:**
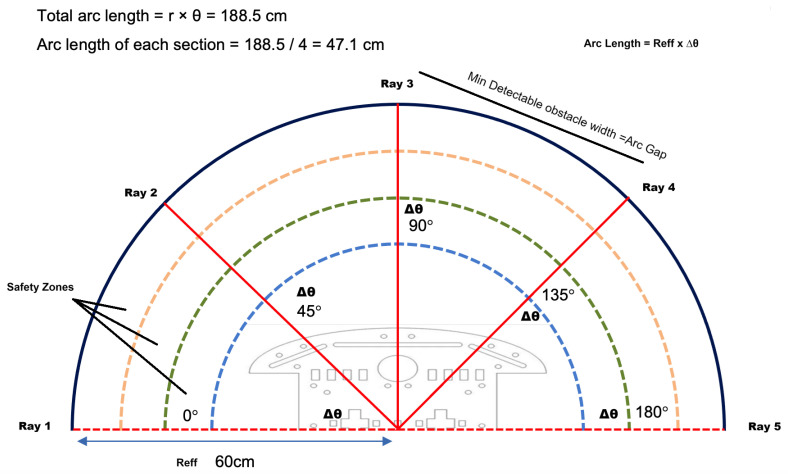
Detection geometry of the sparse LiDAR scan. An illustrative 5-ray example distributed across a 180° field of view creates an angular spacing Δθ between adjacent rays. At the nominal design range of 60 cm, the total semicircular arc is π×60≈188.5 cm, which is divided into N−1=4 equal sections of 188.5/4≈47.1 cm each. This arc length per section corresponds to the minimum detectable obstacle width $W_{\min}$ at that range for this configuration. The effective sensing range $R_{\mathrm{eff}}=57.17$ cm is used in all design calculations after subtracting the measured sensor error. Obstacles smaller than the arc gap may fall between adjacent rays and remain undetected.

Figure 3 illustrates this detection geometry. Because the figure is schematic, it uses an illustrative 5-ray example rather than the final 7-ray operating configuration.

The annotated arc values in Figure 3 can be verified directly from the geometric model. At the nominal design range of 60 cm, the total semicircular arc spanning 180° is π×60≈188.5 cm. With a 5-ray illustrative example (N=5), this arc is divided into N−1=4 equal sections, giving 188.5/4≈47.1 cm per section. This arc length corresponds to the minimum detectable obstacle width at that nominal range for the illustrated configuration, as defined by Equation (7). Note that in all quantitative design calculations, the effective sensing range $R_{\mathrm{eff}}=57.17$ cm is used in place of the nominal 60 cm, to account for measured sensor error.

The minimum detectable object width also depends on distance. As the obstacle moves farther away, the physical gap between adjacent rays becomes larger. This means that an object that can be detected in a near zone may not be detected in a farther zone using the same sparse scan. On the current platform, the forward-sensing field was divided into proximity zones. The main idea was to directly link beam count, object size, and zone distance.

Table 1 makes an important design point clear: a fixed sparse scan does not have a fixed detection limit everywhere. Its detection limit changes with distance. As a result, the beam count, the zone boundaries, and the minimum blocking obstacle width must be designed together.

The detection model and latency model can be combined to justify the selected sparse configuration. The geometric requirement from Equation (9) gives N≥6.99, so N≥7 is required for the target obstacle class at the chosen effective range. At the same time, the sparse scan should remain short enough to preserve a useful navigation update rate. For the present hardware, measured quick-scan times show that N=9 and N=11 remain safe but increase latency without improving detection performance for the target obstacle class, whereas N=5 remains too sparse to provide reliable detection. Thus, the smallest configuration that satisfies the practical detection requirement while preserving a favourable timing margin is N=7.

For the platform studied here, the 7-point sparse scan is the minimum practical configuration that satisfies the design detection requirement for the target obstacle class and achieves collision-free navigation in the reported indoor experiments. Equation (12) shows that the geometric threshold lies at N≈6.99, so N=5 does not satisfy the target detection requirement and N=7 is the smallest odd integer that does.

The quick scan is only useful if it also fits within the robot’s stopping margins. The sparse scan reduces decision latency according to Equation (14), but safe operation also depends on the motion-coupled constraint in Equation (18). The practical implication is that beam count, cycle time, and allowable speed must be selected together rather than independently. This makes the 7-point scan important both operationally and geometrically. A sparser scan may be faster but unsafe because of missed detections; a denser scan may be safer geometrically but unnecessarily restrictive in terms of reaction time and forward motion. The selected operating point therefore reflects a combined detectability–latency–safety trade-off rather than a single criterion.

The sparse forward scan is used to update the obstacle state before the robot advances to the next mapping step. After one horizontal dense sweep is completed at a given tilt angle, a sparse forward scan is executed. The robot then moves forward while the next horizontal sweep is acquired at the following tilt angle. Let $T_{h}$ denote the time required for one horizontal dense sweep, $T_{s}$ the time required for one sparse forward scan, and $T_{d}$ the decision and control update time. The total time $T_{c}$ between two consecutive sparse obstacle updates is

(13) $$T_{c}=T_{h}+T_{s}+T_{d}.$$

The quick-scan time for a sparse scan with *N* points is denoted $T_{q}\left(N\right)$, while the dense baseline scan time is denoted by $T_{\mathrm{full}}$. The latency reduction factor is

(14) $$\eta =\frac{T_{\mathrm{full}}}{T_{q}\left(N\right)}.$$

For the present platform, the measured step time was approximately 500 ms, so the sparse-scan time is well approximated by

(15) $$T_{q}\left(N\right)\approx 500N\;\mathrm{ms}.$$

If the robot moves forward at speed *v*, then the distance travelled during one scan cycle is

(16) $$d_{f}=vT_{c}.$$

For safe operation, this distance must remain less than the available detection and braking distance. Let $d_{\mathrm{safe}}$ denote the available safe forward distance and let $d_{\mathrm{brake}}$ denote the braking distance of the robot. A conservative safety condition is therefore

(17) $$d_{f}+d_{\mathrm{brake}}\leq d_{\mathrm{safe}}.$$

Substituting Equation (16) into Equation (17) gives the maximum allowable robot speed,

(18) $$v_{\max}\leq \frac{d_{\mathrm{safe}}-d_{\mathrm{brake}}}{T_{c}}.$$

In practice, the horizontal sweep dominates the cycle time, so the forward distance travelled during one interleaved update can often be approximated by

(19) $$d_{f}\approx vT_{h}.$$

This approximation is useful when selecting the sparse-scan look-ahead distance. The sparse scan should cover at least the distance the robot may travel during the next horizontal sweep, together with braking distance and an additional safety margin. This requirement can be written as

(20) $$R_{\mathrm{lookahead}}\geq vT_{h}+d_{\mathrm{brake}}+d_{\mathrm{margin}},$$

where $R_{\mathrm{lookahead}}$ is the forward range covered by the sparse scan and $d_{\mathrm{margin}}$ is an additional safety allowance.

## 4. Robotic Platform, Experiment Results and Discussion

### 4.1. Robotic Platform, Experimental Setup, Scan Timing, and Sensor Accuracy

**Figure 4:**
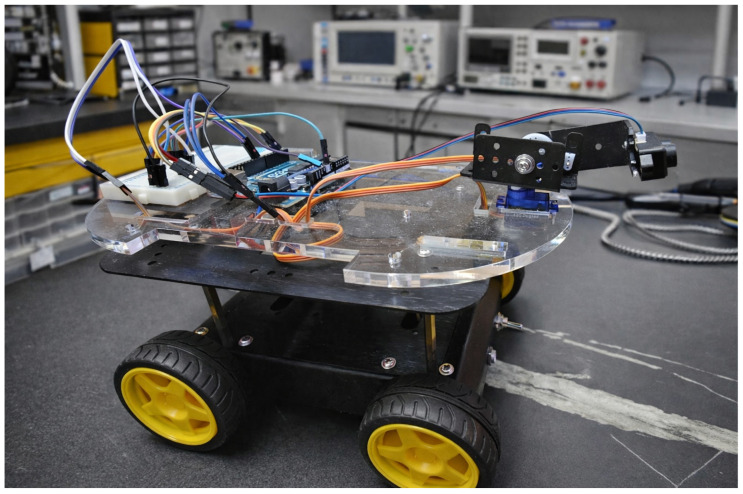
Experimental robotic platform equipped with a servo-actuated LiDAR scanning unit. The sensor is mounted on a pan–tilt mechanism that performs horizontal sweeps from 0° to 180° while incrementally adjusting the vertical tilt angle from 0° to 40°. This configuration enables dense 3D environmental scanning while supporting the interleaved sparse scan used for obstacle detection.

The sensing framework was implemented on a differential-drive mobile robot. The platform includes a LiDAR sensor, a servo scanning unit, an embedded controller, a motor driver, and a battery pack, details of which are given in Table 2. The hardware platform is shown in Figure 4.

**Figure 5:**
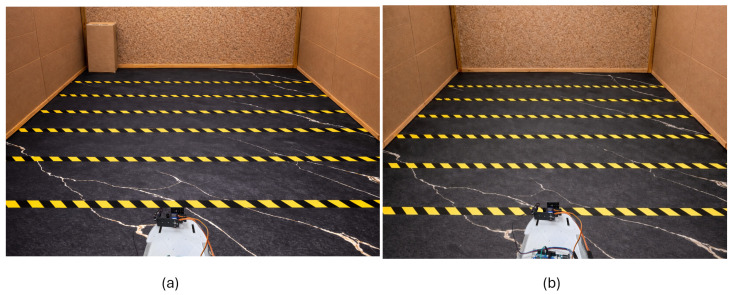
Experimental environment used for evaluating the proposed LiDAR scanning system. The experiments were conducted in a small indoor test arena (1.6m×2.0m) with distance markers placed along the floor to provide reference intervals. The mobile robot equipped with a servo-actuated LiDAR scanner was positioned at the starting location, while a rectangular cardboard obstacle was placed within the LiDAR’s field of view. (**a**) Experimental setup with an obstacle present. (**b**) Baseline environment without an obstacle.

The experiments were carried out in a real indoor test area with an effective footprint of approximately 1.6m×2.0m, as shown in Figure 5. This footprint was chosen to replicate a typical small indoor workspace such as a corridor section or a cluttered room, where forward sensing range is limited and reactive obstacle avoidance is most critical. The constrained area also ensures that the robot operates consistently within the effective sensing range of 60 cm, allowing the detection model to be validated under its design conditions.

The main detection-rate test used a rectangular obstacle 30 cm wide, 30 cm deep, and 50 cm high, positioned at 60 cm from the sensor origin. The 30 cm width was selected to represent a blocking obstacle of comparable scale to the robot body width, corresponding to the minimum obstacle size that must be reliably detected to prevent a collision on the current platform. For each value of *N*, 100 trials were run with randomised angular placement. Additional worst-case tests placed the obstacle between adjacent rays.

During interleaved operation, the robot advanced approximately 5 cm after each sparse scan cycle. Because LiDAR acquires measurements sequentially as the platform moves, forward motion can introduce point-cloud distortion if successive scan rows are combined without pose correction. To mitigate this effect during mapping, each LiDAR point was transformed using the estimated robot displacement between scan cycles, producing a motion-compensated reconstruction [10,21,22].

Detection results are reported both as observed proportions and as two-sided 95% Wilson confidence intervals computed from the trial counts [42]. These intervals are included to make the uncertainty of finite-sample detection rates explicit, especially for the threshold region between the under-sampled and fully reliable configurations.

**Figure 6:**
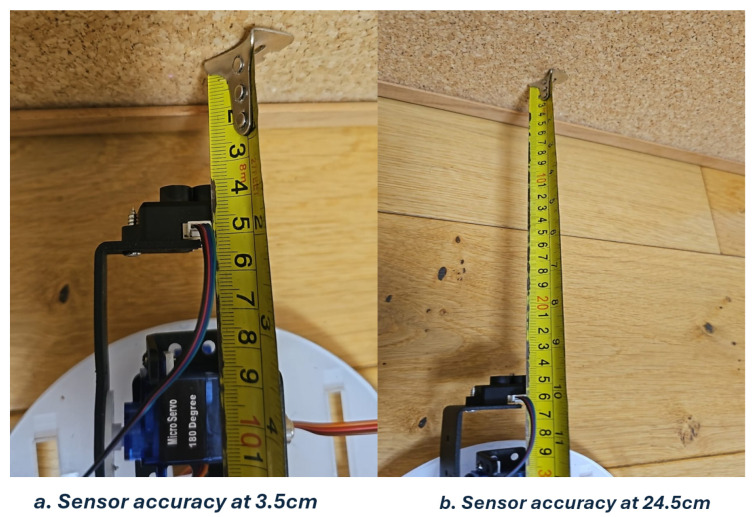
Sensor accuracy test results at ground-truth distances of 3.5 cm and 24.5 cm, showing measured readings from 50 trials at each distance.

The minimum stable inter-scan delay found during step-response testing of the servo actuator was 500 ms. Sensor accuracy was estimated at two known target distances, 3.5 cm and 24.5 cm, with 50 readings taken at each distance as shown in Figure 6. The per-distance mean absolute error (MAE) values are summarised in Table 3. Signal strength is a dimensionless intensity count returned by the sensor firmware. A conservative error bound of ε=2.83 cm was derived from the full 100-trial dataset and used when defining the effective sensing range; this bound exceeds both per-distance MAE values to ensure that the design does not underestimate sensor error. The largest individual errors occurred at the shortest distance (3.5 cm), consistent with time-of-flight resolution limits at very close range.

The scan-time and navigation results are summarised in Table 4. The dense baseline scan took 21.7 s. The 7-point quick scan took 3.512 s.

Substituting these measured values into Equation (14) gives

(21) $$\eta =\frac{T_{\mathrm{full}}}{T_{q}\left(7\right)}=\frac{21,700}{3512}\approx 6.18,$$

rounded to 6.2 throughout this paper.

### 4.2. Detection Validation and Safety Constraints

Table 5 shows the outcome of the 100-trial experiment performed at 60 cm range using a 30 cm wide obstacle. The purpose of this experiment was to test the threshold behaviour predicted by the geometric model.

**Figure 7:**
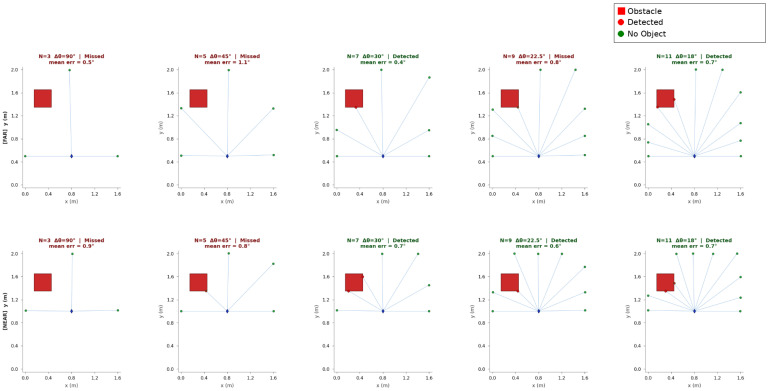
Effect of sparse LiDAR scan resolution on obstacle detection. Different numbers of scan rays (*N*) and corresponding angular spacing (Δθ) are shown for two distance scenarios: the (**top**) row shows the far scenario with the obstacle at 60 cm range, and the (**bottom**) row shows the near scenario with the obstacle at 30 cm range. The blue rhombus marker (⧫) indicates the position of the target obstacle within the scan field. Larger angular spacing results in gaps between adjacent rays, increasing the likelihood of missing the obstacle, while smaller spacing increases the likelihood of detection.

A 3-point scan is too sparse. A 5-point scan improves the situation, but it still misses the target too often. From 7 points onward, the target obstacle is detected in every trial. This matches the prediction from the geometric model and supports the choice of the 7-point scan for the platform used here. Figure 7 provides a visual comparison of these sparse-scan configurations.

**Figure 8:**
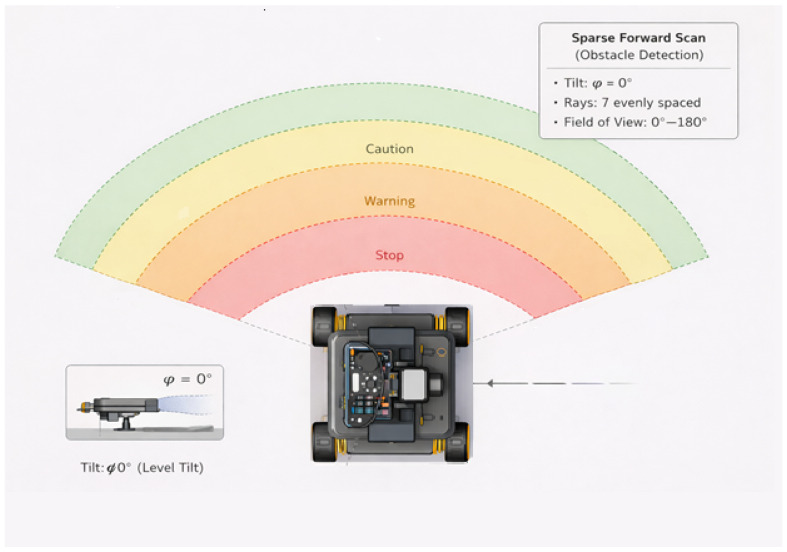
Zone-based safety regions used in the ISDS navigation framework. Colours indicate zone classification: green = safe (>60 cm), yellow = caution (>40 cm), orange = warning (>20 cm), red = stop (≤20 cm). The safe, caution, warning, and stop zones define the robot’s response behaviour as a function of the measured obstacle distance. The sparse 7-point forward scan at tilt ϕ = 0° provides the range readings used to classify each zone.

The quick scan is only useful if it also falls within the robot’s stopping margins. Table 6 summarises the zone thresholds and safety margins used in the study. Figure 8 shows the different zones.

All active zones remain above the braking-distance threshold. The warning zone is the tightest case.

### 4.3. Navigation Trial and Dense Mapping

The horizontal dense sweep also constrains motion because the robot can only move safely if the next obstacle update arrives before it has covered too much ground. Table 7 links the measured horizontal sweep time to the maximum allowable robot speed using the present safety margin.

Table 8 shows the forward travel distance for several robot speeds using the 7-point scan cycle time.

Table 9 compares the maximum allowable speed for different scan configurations using the same safety margin.

**Figure 9:**
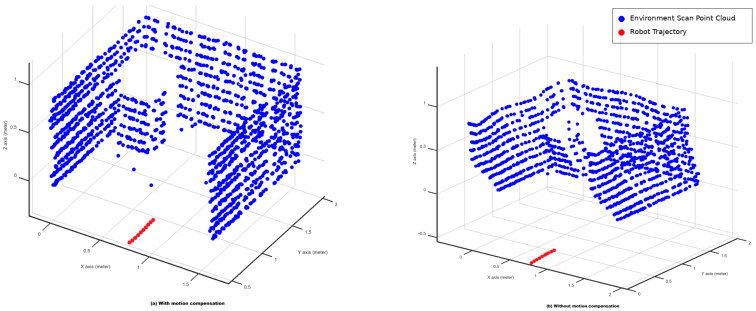
Effect of robot motion compensation on the reconstructed 3D LiDAR point cloud while the robot moves approximately 5 cm between successive scan cycles. (**a**) Reconstruction after applying motion compensation, resulting in improved spatial consistency; the red dots indicate the sequence of estimated robot positions used for pose-based correction, and their diagonal pattern reflects the approximately 5 cm forward displacement applied between successive scan cycles. (**b**) Reconstruction without motion compensation, showing distortion caused by platform movement.

These results explain why the 7-point scan is important both operationally and geometrically. The dense scan mode was used to reconstruct a point cloud of the environment. The mapping part of the system is not the main novelty of the paper, but it is still important because it shows that the platform can build a useful spatial representation while using the sparse scan for navigation. During these experiments, the robot moved forward by approximately 5 cm between successive sparse scan cycles. Figure 9 compares the reconstructed point clouds obtained with and without motion compensation.

In Figure 9a, the red dots indicate the sequence of estimated robot positions used for pose-based correction. Their diagonal pattern reflects the approximately 5 cm forward displacement applied between successive scan cycles. This correction does not implement a full SLAM pipeline; it applies a rigid pose offset per scan row to reduce the inter-row misalignment caused by platform motion.

### 4.4. Discussion

The strongest outcome of this work is that the sparse scan can be chosen from a simple geometric rule and then checked against real timing and navigation results. The 7-point scan was not chosen because it looked good in one plot. It came from the platform dimensions, the effective sensing range, and the target blocking obstacle width, and the experiments then confirmed that choice.

The results also show why the scope of the claims matters. The method works very well for the target 30–40 cm blocking obstacle class in a static indoor scene. It does not guarantee reliable detection of a 5 cm pole, and it performs less well for dynamic obstacles that can move through the field of view within a single scan interval. That is a consequence of the assumptions built into the scan design.

Another important point is that reaction time matters just as much as spatial coverage on this kind of platform. A dense scan provides more environmental detail, but it causes the robot to wait too long before the next motion decision. The sparse scan solves that problem, but only if it is still dense enough to remain safe. That is why the best result in this paper is not the fastest scan. It is the fastest scan that still meets the detection requirement.

The zone analysis adds another layer to that conclusion. The same sparse beam count does not give the same minimum detectable object size everywhere. As distance increases, the physical gap between adjacent rays widens. That means zone boundaries, beam count, and minimum blocking obstacle width must be designed together.

The forward-motion analysis makes the method more complete. It shows that scan timing and robot speed cannot be treated separately. Even a geometrically safe sparse scan becomes operationally unsafe if the robot travels too far before the next obstacle update. In the current setup, the 7-point scan is the best compromise because it remains safe both in terms of object detectability and practical forward motion.

The motion-compensation comparison in Figure 9 adds an important practical result. Because the robot advances by approximately 5 cm between successive scan cycles, directly aggregating raw scan rows produces visible distortion in the reconstructed point cloud. After applying pose-based correction, the overall structure becomes more spatially consistent. This does not turn the system into a full SLAM pipeline, but it does improve the utility of the dense scan for compact 3D reconstruction [21,22,23,24].

The mapping result should also be interpreted carefully. This paper does not introduce a new SLAM algorithm or a new dense reconstruction method. The mapping component is included to show that periodic dense sweeps can still provide useful spatial information, while the sparse forward scan handles obstacle detection and navigation.

A direct numerical comparison with related scanning methods is not straightforward because they operate on different hardware platforms, sensor modalities, and environments. Dwijotomo et al. [38] addressed adaptive scan scheduling within the Cartographer SLAM pipeline using multi-beam LiDAR, focusing on computational cost rather than mechanical latency. Tasneem et al. [39] proposed adaptive foveated scanning for depth cameras, targeting non-uniform spatial allocation rather than embedded obstacle detection. Peters and Knoll [40] considered actuated 3D sensors for self-calibration tasks. None of these works link beam count, mechanical scan latency, and safe robot forward motion in a unified embedded-platform framework. The primary distinction of the proposed ISDS method is its explicit geometric design procedure that connects sensing density, detection reliability, and motion safety on a cost-constrained, mechanically actuated single-beam platform. Table 10 summarises these differences qualitatively.

There are several limitations that should be noted clearly. The system uses mechanical scanning, which limits its operating speed. The servo motor used in this study operates at a minimum step delay of 500 ms, which directly caps the maximum scan update rate; higher-torque or lower-inertia actuators could reduce this bottleneck and increase the obstacle-update frequency. The tests were carried out indoors on a single differential-drive platform and with a specific robot geometry. The validation is therefore performed in a controlled static environment, which raises questions about scalability to more dynamic or outdoor scenarios. While the geometric model holds for any mechanically actuated single-beam LiDAR, the specific timing constants ($T_{q}$, $T_{h}$) and safety margins are platform-dependent and would require re-calibration for different robot geometries or actuator configurations.

The direct experimental comparisons were made against internal baselines, not against external algorithms tested on the same hardware. The present formulation is therefore configuration-specific rather than universally optimal. The experiment area is also deliberately small, so the paper should be read as a constrained embedded-platform study rather than as a claim of broad-environment deployment readiness.

The system also has reduced effectiveness against dynamic obstacles that can move through the field of view within a single scan interval. Combining the sparse LiDAR scan with a complementary sensing modality, such as an optical flow sensor or event camera, could provide inter-scan dynamic detection capability and is identified as a direction for future work.

## 5. Conclusions

This paper presented an Interleaved Sparse–Dense Scanning (ISDS) method for a low-cost embedded robot equipped with a single servo-actuated LiDAR sensor. The central goal was to reduce obstacle-response latency without abandoning environmental mapping.

A geometric model was used to link beam count, sensing range, and minimum detectable obstacle width, yielding a 7-point sparse forward scan for the platform studied here. Experiments confirmed that this configuration achieved full detection of the target 30–40 cm obstacle class at 60 cm range and reduced decision latency by 6.2 times compared with sequential full-scan operation.

The key finding is that the best configuration is not the fastest scan, but the fastest scan that still satisfies the geometric detection requirement. The zone analysis further shows that beam count, zone boundaries, and minimum detectable obstacle width must be designed jointly, not independently. The motion analysis adds that scan timing and robot speed are also coupled: even a geometrically safe sparse scan becomes operationally unsafe if the robot travels too far before the next obstacle update. For the current hardware, the 7-point ISDS scan is the best compromise across all three criteria: detection reliability, response latency, and forward-motion safety.

Future work will focus on speed-aware scan scheduling with integrated motion compensation, enabling the scan pattern to adapt online to vehicle velocity and manoeuvring conditions. Extending the validation to more diverse environments and incorporating complementary sensing modalities for dynamic obstacle detection are also planned.

## Figures and Tables

**Table 1:** Comparison of scan-point count and minimum detectable object size at different distances.

No. of Scan Points	Object Size at 60 cm (cm)	Object Size at 50 cm (cm)	Object Size at 40 cm (cm)	Object Size at 30 cm (cm)
4	94.25	78.50	62.85	47.10
6	47.13	39.25	31.43	23.55
7	37.70	31.42	25.13	18.85
8	31.42	26.17	20.95	15.70
10	23.56	19.63	15.71	11.78
12	18.85	15.70	12.57	9.42
14	15.71	13.08	10.48	7.85
16	13.46	11.21	8.98	6.73
18	11.78	9.81	7.86	5.89
20	10.47	8.72	6.98	5.23
22	9.43	7.85	6.29	4.71
24	8.57	7.14	5.71	4.28
26	7.85	6.54	5.24	3.93
28	7.25	6.04	4.83	3.62
30	6.73	5.61	4.49	3.36

**Table 2:** Hardware configuration of the experimental robotic platform.

Component	Description
LiDAR sensor	Low-cost time-of-flight range sensor used for distance measurement
Servo motor	Scanning actuator used to position the sensor during sparse and dense scans
Embedded controller	Onboard control unit used for scan scheduling, obstacle logic, and motion control
Motor driver	Interface between controller and drive motors
Drive system	Differential-drive wheeled base
Power supply	Rechargeable battery system for mobile operation

**Table 3:** Sensor accuracy results at two ground-truth distances (50 trials each).

Ground Truth (cm)	Trials	Mean Reading (cm)	MAE (cm)	Signal Strength (Counts, Approx.)
3.5	50	1.0	2.50	∼8073
24.5	50	22.3	2.17	∼13046

**Table 4:** Latency and navigation performance.

Configuration	*N*	$T_{q}$ Theory (ms)	$T_{q}$ Measured (ms)	η	Comment
Full scan baseline	37	18,600	21,700	1.0×	Too slow for reactive use
ISDS sparse scan	5	2500	2612	8.3×	Fast, but missed obstacles
ISDS sparse scan	7	3500	3512	6.2×	Best overall result
ISDS sparse scan	9	4500	4498	4.8×	Safe, but slower than needed
ISDS sparse scan	11	5500	5503	3.9×	Diminishing return

**Table 5:** Arc-length analysis and measured detection rates.

*N*	α (°)	Arc at 60 cm (cm)	$W_{\min}$ at $R_{\mathrm{eff}}$ (cm)	Detections	Detection Rate (95% CI)	Condition Satisfied
3	90.0	94.2	89.8	23/100	23% (15.8–32.2)	No
5	45.0	47.1	44.9	71/100	71% (61.5–79.0)	No
7 ^†^	30.0	31.4	29.9	100/100	100% (96.3–100.0)	Yes
9	22.5	23.6	22.5	100/100	100% (96.3–100.0)	Yes
11	18.0	18.8	18.0	100/100	100% (96.3–100.0)	Yes

^†^ Selected operating configuration used in all subsequent navigation experiments.

**Table 6:** Zone safety validation for the selected configuration.

Zone	Threshold	$d_{z}-\varepsilon$ (cm)	$d_{\mathrm{brake}}$ (cm)	Safety Margin (cm)	Pass
Safe	>60 cm	57.17	11.25	45.92	Yes
Caution	>40 cm	37.17	11.25	25.92	Yes
Warning	>20 cm	17.17	11.25	5.92	Yes
Stop	≤20 cm	E-stop	–	–	Yes

**Table 7:** Effect of horizontal scan step size on sweep time and maximum allowable forward speed.

Horizontal Step	Points per Sweep	Measured Sweep Time $T_{h}$ (s)	Approx. $v_{\max}$ (m/s)	Comment
30°	7	3.6	0.128	Fast enough for motion, but coarse for dense mapping
20°	10	6.0	0.077	Better spatial detail, lower motion envelope
15°	13	8.0	0.057	Moderate detail, slower update
10°	19	15.0	0.031	Good detail, but restrictive for reactive motion
5°	37	21.6	0.021	Best dense detail in this study, but too slow for frequent motion updates

**Table 8:** Forward travel distance during one interleaved scan cycle for different robot speeds.

Robot Speed (m/s)	Cycle Time $T_{c}$ (s)	Distance per Cycle $d_{f}$ (cm)	With 11.25 cm Braking (cm)	Assessment
0.02	3.51	7.0	18.3	Safe in present setup
0.05	3.51	17.6	28.9	Acceptable with margin
0.08	3.51	28.1	39.4	Borderline for tight indoor spaces
0.10	3.51	35.1	46.4	Requires larger look-ahead margin
0.15	3.51	52.7	64.0	Unsafe for current 60 cm detection range

**Table 9:** Effect of scan cycle time on maximum allowable robot speed.

Configuration	Cycle Time (s)	$d_{\mathrm{safe}}-d_{\mathrm{brake}}$ (cm)	$v_{\max}$ (m/s)	Comment
5-point sparse scan	2.61	45.92	0.176	Faster, but unsafe due to missed detections
7-point sparse scan	3.51	45.92	0.131	Best balance for this platform
9-point sparse scan	4.50	45.92	0.102	Safe, but slower operating envelope
Full dense scan only	21.70	45.92	0.021	Too slow for reactive navigation

**Table 10:** Qualitative comparison of the proposed ISDS method with related scanning approaches.

Method	Sensor Type	Latency Focus	Embedded	Geometric Model	Motion Safety Link	Single-Beam
Dwijotomo et al. [38]	Multi-beam LiDAR	Partial	Not explicit	Not explicit	Not explicit	Not explicit
Tasneem et al. [39]	Depth camera	Primary focus	Limited	Not explicit	Not explicit	Not explicit
Peters & Knoll [40]	Actuated 3D	Not explicit	Not explicit	Not explicit	Not explicit	Not explicit
**Proposed ISDS**	Single-beam ToF	Primary focus	Yes, explicit	Yes, explicit	Yes, explicit	Yes, explicit

**Bold** row indicates the method proposed in this paper.

## Data Availability

Data supporting the reported results are available from the corresponding author upon reasonable request.
